# *In vitro* transfection of anti-tumor miR-101 induces BIM, a pro-apoptotic protein, expression in acute myeloid leukemia (AML)

**DOI:** 10.17179/excli2017-721

**Published:** 2017-11-27

**Authors:** Narges Nikoonahad Lotfabadi, Homa Mohseni Kouchesfahani, Mohammad Hasan Sheikhha, Seyed Mehdi Kalantar

**Affiliations:** 1 Department of Animal Biology, Faculty of Biological Sciences, Kharazmi University, Tehran, Iran; 2 Biology Department, Faculty of Sciences, Science and Arts University, Yazd, Iran; 3 Reproductive & Genetic Unit, Research and Clinical Center for Infertility, Shahid Sadoughi University of Medical Sciences, Yazd, Iran; 4 Biotechnology Research Center, International Campus, Shahid Sadoughi University of Medical Science, Yazd, Iran

**Keywords:** AML, miR-101, BIM, apoptosis

## Abstract

Acute myeloid leukemia (AML) frequently relapses after initial treatment, though it is possible that drug resistance occurs. Hence, it seems necessary to develop novel therapies such as gene therapy specifically via miRNA transfection. MicroRNA-101 has been considered as a tumor suppressor in different types of cancer. It is demonstrated that exogenous miR-101 transfection is associated with decreased viability in AML in this paper. Besides, the increase of pro-apoptotic protein BIM expression in both mRNA and protein level has been illustrated. The recent findings provide an insight into the novel function of miR-101 in AML by activating BIM as an important mediator in intrinsic apoptosis pathways. Generally, miR-101 has been considered as a therapeutic target in our data and might have a valuable role in AML.

## Introduction

Acute Myeloid Leukemia (AML) is a comparatively rare cancer that appears in people with average age of 60 years. Its incidence among younger group of people is 2-3 cases among 100000 patients. It increases to 13-15 cases per 100000 individuals, among the age of 70-80 years; *i.e.* the incidence of this disease increases by age (Altucci et al., 2005[1]; Burnett et al., 2011[6]). AML is a hematopoietic stem cell malignant tumor of non-lymphoid category and a genetically clonal and heterogeneous disorder, diagnosed by the accumulation of somatically acquired genetic disorders. These transformations change the natural mechanisms of renewal, proliferation and differentiation (Altucci et al., 2005[1]; Marcucci et al., 2011[26]). There is significant amount of information that confirm that AML includes a disease or a set of diseases, which are severely heterogeneous from the aspect of morphology, cytochemistry of leukemic population, immunophenotype, cytogenetic and molecular disorders that originate from mutation or over-expression of some genes (Burnett et al., 2011[6]). In other words, AML is a group of hematopoietic malignancies, diagnosed by the retarded growth and the maturation of myeloid cells in bone marrow and blood (Cammarata et al., 2010[7]).

At present, chemotherapy is the sole treatment of AML in young patients, in which different chemotherapeutic agents of anthracycline family are combined. While, for the patients of over 60 years age, combined chemotherapy is not possible, hence there has developed a huge tendency towards modern therapeutic approaches (Burnett et al., 2011[6]). Nowadays, various efforts are being carried out across the globe to develop modern technologies in order to prevail over inherent deficiencies of chemotherapy and combat different cancers without causing damage to normal cells (Del Burgo et al., 2014[10]).

Gene therapy is a modern therapeutic approach that can potentially be able to decrease protein or gene expression because of its efficient effects (Nordling-David and Golomb, 2013[27]). It had been used in many diseases such as cancer, AIDS and cardiovascular disorders. Among medical therapeutic procedures, this approach is possible through genetic reagent insertion into patients' special cells, where encoded proteins are produced (Jayakumar et al., 2010[17]). Gene therapy includes the transfection of nucleic acid-based compartments such as; plasmids, antisense oligonucleotides, and different types of RNAs to nucleus or cytosol that are able to be effective to decrease the gene expression that is involved in disease, both *in vivo *and* in vitro* conditions (Nordling-David and Golomb, 2013[27]).

Today, among RNAi therapeutic candidates, microRNAs have been highly regarded. Synthetic miRNA transmission to cells as a constitution that can imitate internal miR performance creates a hopeful method for cancer treatment. miRNAs target several cellular processes and therefore have broad effects toward modern methods (Cortez et al., 2014[8]).

miRNAs are a group of small endogenous noncoding RNAs that have 18-22 nucleotides, expanded among animals and plants. Several studies have shown the importance of these structures in protection of cellular homeostasis (Ji et al., 2017[18]). Researchers have proved the role of miRNAs in a vast scope of biological processes like carcinogenesis and act as key controllers in gene expression. The miRNA expression has been found to be very irregular in cancerous cells (Peng and Croce, 2016[28]; Wang et al., 2015[33]). In fact, miRNAs are functional RNAs that cause gene-expression silencing by targeting the mRNA. Based on miRNA's important roles in oncogenesis, these structures have been assessed as diagnostic biomarkers, oncogenic factors, or tumor suppressors, having therapeutic potentials. miRNAs are generally related to different stages of tumor progression such as; proliferation, metastasis and invasion, angiogenesis, apoptosis and drug/radiation resistance in diseased cells (Wang et al., 2015[33]). Therefore, it can be noted that the acquired knowledge about down-regulation and loss of expression of miRNA provides new opportunities for therapeutic methods. Application of miRNAs as a single therapeutic reagent or as a combination therapy in conventional treatment methods probably presents technical advantages beyond other methods. miRNAs are small molecules and less antigenic toward peptides or protein-encoded genes, so they can function potentially better than other biological moieties (Soriano et al., 2013[31]). 

miR-101 is a tumor suppressor that neutralizes tumor progression and development by down-regulating some oncogenes. It is proved that miR-101 can prevent clonal cancer cell formation *in vitro* and hinder the tumor growth *in vivo*. It is also able to sensitize some types of cancer cells towards apoptosis. Mcl-1 was determined as a functional target for miR-101. Decreased miR-101 expression has been observed in several kinds of cancers (Su et al., 2009[32]; Xu et al., 2014[34]).

Mitochondrial programmed cell death pathways are controlled by Bcl-2 superfamily that consists of several pro-apoptotic and anti-apoptotic proteins. This group is characterized by conserved four BH domains. While, anti-apoptotic proteins such as Bcl-2, Bcl-XL, Mcl-1, Bcl-w have four domains of BH type (BH 1-4). The pro-apoptotic members of this family such as; Bax and Bak have only three domains of BH type (1-3). Another group of proteins, having only BH3 domain (BH3- only), are Bid, Bad, Noxa, Puma, Bik/NBK, BIM isoforms and other members (Plötz et al., 2013[29]). BIM protein at first connect to anti-apoptotic proteins and then allow multi-domain pro-apoptotic proteins like Bak and Bax to form channels on mitochondrial membrane and result in cytochrome-c release and onset of apoptosis (Gogada et al., 2013[12]). RNAi-mediated suppression of BIM suppresses cell death (Kawabata et al., 2012[19]). 

Because of the importance of apoptotic resistance in oncogenesis, tumors necessarily show resistance towards apoptosis (Llambi and Green, 2011[23]). Apoptotic induction provides a supportive mechanism in protection toward carcinogensis by the means of transformed cell omission. So imperfect apoptotic program for tumor cells works as a feature for them and finally causes resistance toward chemotherapy (Plötz et al., 2013[29]). Consequently, application of gene-based drugs such as apoptotic inducing miRNA can increase hopes to cure several kinds of cancers. 

By considering the importance of miRNA as a therapeutic factor in the process of gene therapy in different cancers, the aim of this research was to assess the effect of miRNA-101 on pro-apoptotic BIM expression at protein level in KG-1 (AML cells) and HBMF-SPH (normal bone marrow cells) cell lines.

## Materials and Method

### Chemicals and reagents

Penicillin, streptomycin, 3-(4,5-Dimethyl-thiazol-2-yl)-2,5-diphenyltetrazolium bromide (MTT), HEPES buffer (10 mM, pH=5.5) and microRNA mimic, has-miR-101 were purchased from Sigma-Aldrich (St. Louis, MO, USA). Dimethyl sulfoxide (DMSO) was purchased from Merck (Germany). RPMI-1640 medium, Dulbecco's modified Eagle's medium (DMEM), trypsin, fetal bovine serum (FBS), and phosphate buffered saline (PBS) were purchased from Gibco Medicago. RevertAid™ First Strand cDNA Synthesis Kit was purchased from Thermo Scientific (USA). RNeasy Mini Kit was purchased from Qiagen (USA). Primary antibody was obtained from Santa Cruz Biotechnology (USA). Lipofectamine 2000 and HRP-conjugated secondary antibody was purchased from Invitrogen (USA). Human KG-1 (AML cell line) and HBMF-SPH (normal human bone marrow cells) cell lines were supplied by the Pasteur Institute, Tehran, Iran. Deionized water was used throughout the experiments and all chemicals were of analytical grade unless otherwise indicated.

### Preparation of miR-101 loaded cationic liposomes and transfection 

Lipofectamine 2000 (Lipo), a widely used commercial agent, was used as a cationic liposome (CL) to introduce miRNA-101 to cells. In order to transfect the miR-101 mimics into cells, lipofectamine 2000 was used essentially as described by the manufacturer's protocol. Final concentration of 25 µM of Lipo/miR-101 was used for transfection into bone marrow cells.

### Cell culture 

KG-1 cells, an AML cell line, and HBMF-SPH cells, a normal bone marrow cell line were seeded in 25-cm^2^ flasks in RPMI-1640 medium supplemented with 10 % FBS, 50 mg streptomycin/ml, 50 mg penicillin /ml at 37 °C in a humidified atmosphere, containing 5 % CO_2_. The cells were used for further experiments after three successful passages.

### Cellular uptake 

The KG-1 cells were seeded at a density of 5×10^5^ cells/well in 6-well plates and incubated for 24 h. Then, the cells were treated with FAM-labeled miRNA alone and Lipo/FAM-labeled miRNA at a suitable concentration into each well to determine the cellular uptake of miRNA. After treatment, the cells were incubated for 3 h, washed thrice with PBS and fixed with 4 % paraformaldehyde solution (Thermo Scientific, USA). The solution of 4',6-diamidino-2-phenylindole (DAPI, 0.125 µg/ml) was used to stain live nuclei in KG-1 cells. To achieve this goal, fixed cells were treated with DAPI for 15 min and the cellular uptake was assessed using fluorescent microscope (Olympus, Japan).

### Cytotoxicity evaluation of miRNA containing CLs against bone marrow cell lines

The cytotoxic effects of bare miR-101 and Lipo/miR-101 on KG-1 and HBMF-SPH cells were assessed using MTT assay. To measure the cytotoxicity, both cell lines were seeded in 96-well plates at a density of 1×10^4^ cells/well. After 24 hours, cells were treated with miR-101 and Lipo/miR-101 and incubated again. To assess the viable cell percentages, 20 µl MTT (5 mg/ml) was added to each well and evacuated after 3 h of incubation and 150 µl DMSO was then added to each well. The proportion of viable cells was measured colorimetrically by microplate reader (EPOCH Microplate Spectrophotometer- synergy HTX, Bio Tek, USA) after 72 hours of incubation at 570 nm wavelength.

### In vitro cell transfection

KG-1 and HBMF-SPH cells were seeded at a density of 1×10^6^ cells/well in 6-well plates in RPMI 1640 medium and incubated overnight. Next day, the cells were incubated with 25 µM Lipo/miR-101 and bare miR-101 for 24 h.

### Quantitative Reverse-Transcriptase Polymerase Chain Reaction (qRT-PCR) analysis

Quantitative RT-PCR analysis was applied to determine the relative expression level of *BIM*. After 24 h treatment, total RNA was extracted from cells, using RNeasy Mini Kit (Qiagen, USA), according to the manufacturer's instructions. Following DNase treatment, RNA concentrations, quality and integrity were determined spectrophotometrically using Nanodrop (Waltham, MA, USA). Total RNA (1 mg) was used to convert RNA into single-stranded complementary DNA using RevertAid™ First Strand cDNA Synthesis Kit (Thermo Scientific, USA), according to the manufacturer's instructions. Then, qRT-PCR was performed using SYBRR Green PCR Master Mix to evaluate the expression level of pro-apoptotic gene,* BIM*. Glyceraldehyde-3-phosphate dehydrogenase (*GAPDH*) was used as an endogenous control. Real time PCR was performed at least thrice for all cell lines. The primers for *BIM* were as follows: forward, 5'-TCTGACTCTGACTCTCGGACTG-3' and reverse, 5'-GGATTACCTTGTGGCTCTGTCTG-3' and the primers for *GAPDH* were as follows: forward, 5'-TGCACCACCAACTGCTTAGC-3' and reverse, 5'-GGCATGGACTGTGGTCATGAG-3'. Data were normalized to the endogenous control and analyzed using the comparative threshold cycle method (2^-∆∆Ct^) to determine the relative expression of *BIM* before and after treatment.

### Western blotting analysis

The BIM protein expression was determined by Western blotting analysis. KG-1 cells were seeded in a 6-well plate at a density of 5×10^5^ cells/well and incubated for 24 h. To evaluate the amount of BIM protein in KG-1 cells, total cell protein was extracted by NP40 lysis buffer after 24h treatment. To prevent protein degradation, phosphatase inhibitors (Sigma, USA) and anti-protease cocktail (Roche, Germany) were used. The concentration of extracted protein was assessed by Qubit Protein Assay Kit (Invitrogen, USA). A total of 40 µg protein/sample was used for gel electrophoresis (on 7.5 % and 12 % SDS-PAGE gel) and the resultant proteins were transferred to nitrocellulose membrane. Blocking was performed in 5 % BSA blocking buffer. Then, the blots were incubated with primary antibody at a dilution of 1:1000 at 4 °C overnight and again incubated with secondary antibody at a dilution of 1:2000. Protein detection was carried out ECL Western Blot Detection Reagents.

### Statistical analysis 

To statistically analyze the data, SPSS software (version 22) was used. Quantitative data are expressed as the mean ± standard deviation (SD). Data were analyzed using Students'*t*-test. P < 0.05 was considered statistically significant.

## Results

### Cellular uptake

**Figure 1:**
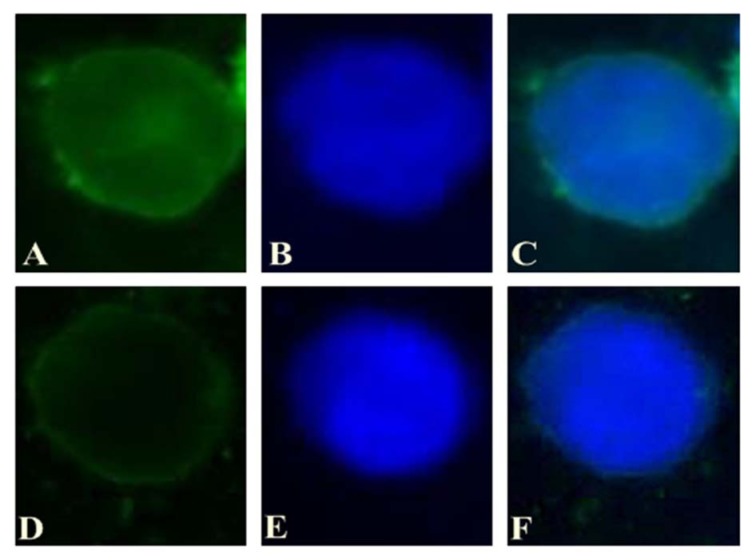
A) The intracellular pattern of Lipo/Fam-labeled miRNA (green) in KG-1 was observed by a fluorescent microscope after incubation for 3 h. B) Nuclei were stained with DAPI prior to observation (blue). C) Merged images. D) The intracellular pattern of bare Fam-labeled miRNA (green) in KG-1 was observed by a fluorescent microscope after incubation for 3 h. E) Nuclei were stained with DAPI prior to observation (blue). F) Merged images

In order to evaluate the ability of gene transfer by lipofectamine 2000 into the cells, KG-1 cells were transfected with Lipo/Fam-labeled miRNA and Fam-labeled miRNA and intracellular study was performed after 3h treatment. *In vitro* cellular uptake of both compositions was assessed using fluorescent microscope (Figure 1(Fig. 1)) (Schnittert et al., 2017[30]). Fluorescence microscopy has shown negligible cellular fluorescence after treatment with bare Fam-labeled miR which indicates its partial transfection into KG-1 cells. While in cells which were treated with Lipo/Fam-labeled miRNA showed remarkably higher fluorescence intensity. As shown in Figure 1(Fig. 1), lipofectamine 2000 could internalize miRNA efficiently into the cells. The results suggest that the cationic lipid-based carrier, such as lipofectamine, can be used to deliver different types of acid nucleic to the cells actively and can be a potent gene delivery system in cancer therapy. 

### Inhibition of AML cells proliferation using miRNA-101

**Figure 2:**
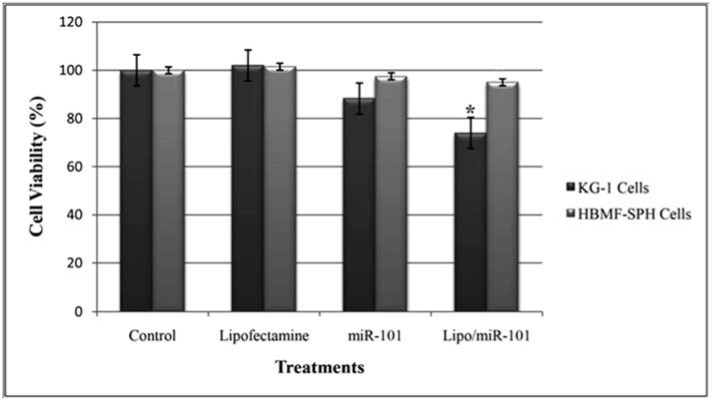
KG-1 and HBMF-SPH cells viability after 72 h of treatment with Lipofectamine, bare miR-101 and Lipo/miR-101. Cell viability decreased significantly in KG-1 cells transfected by Lipo/miR-101 as compared to other groups (p<0.05). (*. The mean difference is significant at the 0.05 level).

**Figure 3:**
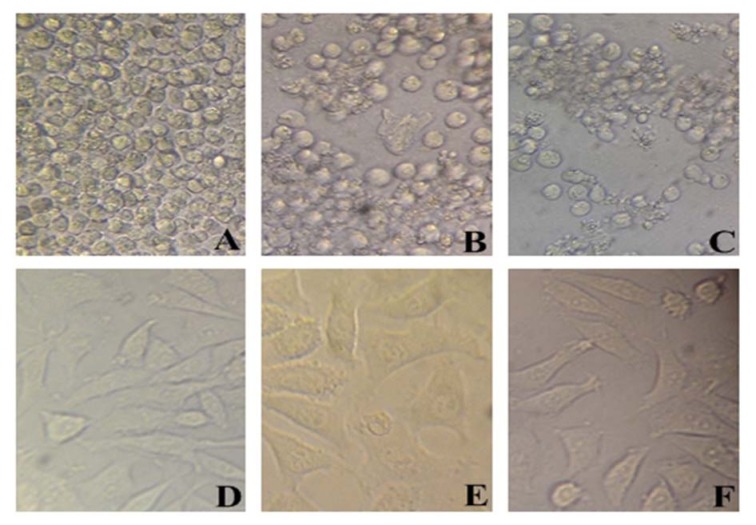
*In vitro* cell viability of KG-1 (A-C) and HBMF-SPH (D-F) cells treated with different interventions. (A) and (D) cells treated with lipofectamine 2000. (B) and (E) cells treated with bare miR-101 for 72 h. (C) and (F) cells treated with Lipo/miR-101 for 72 h. Results showed significant difference between miR-101 and Lipo/miR-101 in KG-1 cell line (*p<0.05*).

To evaluate cytotoxicity of miR-101, MTT assay was used with Lipo/miR-101, naked miR-101 and lipofectamine as treatments in KG-1 and HBMF-SPH cells. Lipofectamine 2000 was used to ensure its non-cytotoxicity. As shown in Figure 2(Fig. 2), lipofectamine 2000, as a cationic lipid-based carrier, showed no anti-proliferative effect in both cell lines. However, KG-1 cells exposed to Lipo/miR-101 and miR-101 showed significant cytotoxicity comparing to HBMF-SPH cells after 72 h treatment (*p<0.05*). In addition, the exposed KG-1 cells to Lipo/miR-101 significantly showed more cytotoxic effects than cells exposed to the bare miR-101 (*p<0.05*). The results of MTT assay have indicated that the Lipo/miR-101 could successfully be entered into the cells as compared to naked miR-101 (Figure 2(Fig. 2)), and specifically exert its anti-proliferative and cytotoxic effects in cancer cells. The results also showed that lipofectamine 2000 as a cationic lipid-based carrier could successfully interact with anionic-charge miR-101 and internalize it into cells effectively (Figure 2(Fig. 2) and Figure 3(Fig. 3)).

### BIM expression is increased by miR-101 transfection 

First, the effect of miR-101 transfection on expression levels of the pro-apoptotic gene, *BIM*, was measured using qRT-PCR. The quantitative real-time PCR analysis displayed that KG-1 cells has very low *BIM *expression level but it is significantly higher in HBMF-SPH cells (*p<0.05*).

**Figure 4:**
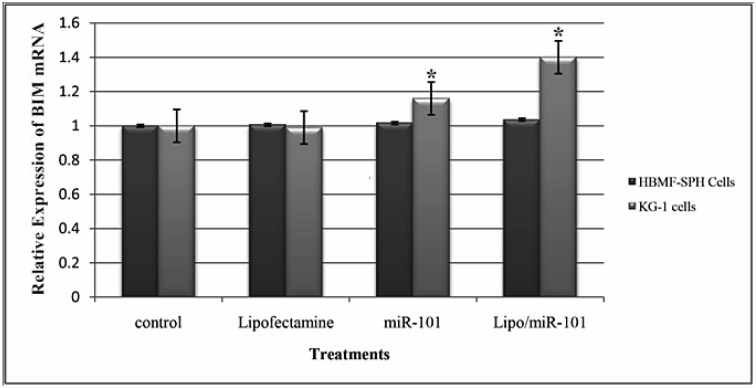
Relative expression level of *BIM* at 24 h after transfection with different treatments in KG-1 and HBMF-SPH cell lines. Results illustrated that relative expression of *BIM* significantly increases after miR-101 transfection (Lipo/miR-101) in KG-1 cell lines (*p<0.05*). (*. The mean difference is significant at the 0.05 level).

As shown in Figure 4(Fig. 4), the *BIM* expression levels were increased in miR-101 and Lipo/miR-101-treated KG-1 and HBMF-SPH cells. *BIM* expression was increased significantly in KG-1 cell line compared to HBMF-SPH cells in miR-101 and Lipo/miR-101 groups (*p<0.05*). Lipo/miR-101 treatment increased *BIM* expression more than other groups in both cells but only in KG-1 cells, its increasing was significant (*p<0.05*). The level of *BIM *expression in KG-1 cells treated with Lipo/miR-101 significantly increased than KG-1 cells exposed to miR-101. So, Lipo/miR-101 treatment caused significant increase in *BIM* expression levels KG-1 cell line (*p<0.05*) (Figure 4(Fig. 4)). However, the results of qRT-PCR indicated that Lipo/miR-101 would notably result in increasing expression of *BIM* in cells (*p<0.05*). These findings showed that the expression of pro-apoptotic gene, *BIM*, is remarkably up-regulated after transfection of miR-101 with cationic lipid-based carrier, lipofectamine 2000. 

**Figure 5:**
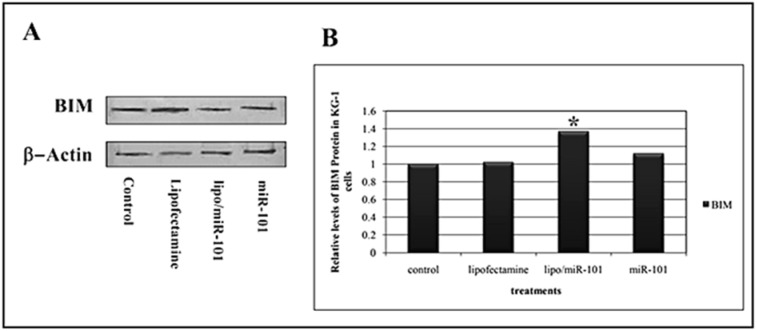
A) Evaluation of the pro-apoptotic BIM protein expression was performed by Western blot analysis at 24 h after transfection. Actin was used as loading markers and shown at the bottom of each blotting membrane analyzed, as indicated. B) Levels of BIM protein in cells treated with different interventions. Results showed that levels of BIM protein significantly increase after miR-101 transfection in KG-1 cells (*p<0.05*). (*. The mean difference is significant at the 0.05 level).

To confirm the initial findings for *BIM* mRNA in KG-1 cell line (AML), Western blot analysis was applied to assess BIM expression at protein level and the correlation of miR-101 and BIM expression was evaluated. The results of Western blotting analysis showed that BIM expression was dramatically increased in treated KG-1 cells (Figure 5(Fig. 5)). As shown in Figure 5B(Fig. 5), Lipo/miR-101 treatment caused more expression in BIM protein in KG-1 cells compared to naked miR-101. Statistical analysis showed that expression of BIM protein was significantly (*p<0.05*) associated with miR-101 transfection with cationic lipid-based carrier. Overall, the results suggested that miR-101 could induce BIM expression and produce BIM protein in KG-1 cells, also induce apoptosis in AML cancer.

## Discussion

A matter of utmost importance is to replace the traditional therapies with conventional cancer treatments such as chemotherapy due to its irreparable side effects (Cross and Burmester, 2006[9]). The recent development in the knowledge of cancer treatments has led to the advanced progress of new therapeutic approaches in cancer therapy, particularly gene therapy. Gene therapy includes any methods to treat a disease by genetic modification in cells. The materials which can be used in gene therapy may be different types of acid nucleic such as plasmid, microRNA or siRNA (Amer, 2014[2]; Husain et al., 2015[16]).

MicroRNA is one of the factors that have proven to play an important role in carcinogenicity. Its involvement in carcinogenesis is a multi-stage process. Studies that featured miRNA overexpression or ablation in different cancers can prove the correlations between miRNAs and carcinogenesis (Ji et al., 2017[18]). In recent years, many of the discovered miRNA regulatory roles have led to an interest in these categories of nucleic acid. In addition to their roles in cellular processes, miRNAs also show considerable potential for diagnostic and therapeutic usages (Baumann and Winkler, 2014[5]). How miRNA enters into the cell, is one of the major challenges in miRNA application as a therapeutic agent. Owing to its charge and size, miRNA can hardly cross the cell membrane. It is necessary to develop carriers with the purpose of gene delivery in order to transfect gene into cells efficiently. Lipofectamine 2000 is one of the commercial reagents which is developed as a cationic lipid-based carrier for gene delivery. It is an effective carrier for miRNA delivery according to previous studies (Bakhshandeh et al., 2012[3]). In the present study lipofectamine 2000 was used as a miRNA carrier. Fluorescent microscopy results confirm that miRNA internalization into cells with lipofectamine 2000 is more efficient than bare miRNA (Figure 1(Fig. 1)). These findings can be referred to Bakhshandeh et al. (2012[3]) and Huang et al. (2015[15]).

The FAM-labeled miRNA and Lipo/FAM-labeled miRNA transfection efficiency was examined by fluorescent microscopy. In agreement with previous findings, current study showed that Lipofectamine 2000 can be used for cancer cells transfection efficiently (Huang et al., 2015[15]).

The results of MTT assay illustrated that the viability of KG-1 cells was significantly decreased in the Lipo/miR-101 group compared to the other groups. These findings imply that miRNA-101 might function as a tumor suppressor in AML. We have noticed that exogenous miR-101 significantly inhibited the cell proliferation in AML (Figures 2(Fig. 2) and 3(Fig. 3)). This is in agreement with the findings of the present study that miR-101 might have a tumor suppressive role in different types of cancer. Xu et al. (2014[34]) indicated that transfection of miR-101 repress proliferation of hepatocellular carcinoma cell lines (Xu et al., 2014[34]). Bao et al. (2016[4]) obtained the same results in gallbladder carcinoma (Bao et al., 2016[4]). Other researchers reported the same results in other types of cancer (Huang et al., 2013[14]; Liu et al., 2017[22]; Luo et al., 2012[25], 2013[24]).

BIM is a pro-apoptotic BH3-only protein of Bcl-2 family that binds to anti-apoptotic proteins; therefore, allows the pro-apoptotic multidomain proteins, Bax and Bak to lead to cytochrome-c release and apoptosis. Pro-apoptotic protein BIM is frequently induced upon treatment with anticancer therapeutics. However, BIM induction-based anticancer therapy includes many limitations such as diversity in controlling systems of BIM in cancer cells (Gogada et al., 2013[12]). It has been suggested that in different malignancies transfection of miR-101 can lead to cell proliferation inhibition and play a consistent suppressive role in tumor development (Gui and Shen, 2012[13]).

In the present study, we evaluated the BIM expression level after miR-101 transfection. In order to assess the relative expression of BIM, a pro-apoptotic member of Bcl-2 superfamily, qRT-PCR and then Western blotting have been done after miR-101 transfection. In this study, we observed a significant overexpression of BIM in both mRNA and protein levels in AML cell line after transfection. It seems that exogenous miR-101 can result in some changes in the expression of anti-and pro-apoptotic members of Bcl-2 family. It has been reported that myeloid cell leukemia sequence 1 (Mcl-1), an anti-apoptotic member of Bcl-2 superfamily, was specified as a direct target of miR-101 (Gui and Shen, 2012[13]). Although another study has indicated that miR-101 can suppress proliferation, migration and invasion, and stem cell-like phenotype of aggressive endometrial cancer cells, at least through targeting EZH2, MCL-1 and FOS. It means that endogenous mRNA and protein levels of these target genes were down-regulated in miR-101-transfected cells (Konno et al., 2014[21]). Glaser et al. (2012[11]) reported that decreased expression of Mcl-1 kills transformed myeloid cells *in vitro* by activating the mitochondrial or intrinsic apoptotic pathway in a BIM-dependent manner (Glaser et al., 2012[11]). Zhang et al. (2011[35]) indicated that down-regulation of EZH2 by miR-101 induces BIM expression, and hence, apoptosis will be increased in non-small cell lung cancer cells (Zhang et al., 2011[35]). In addition, Kim et al. (2017[20]) suggested that miR-101 can sensitize tumor cells apoptosis by up-regulation of BIM expression in an EZH2-dependent manner, indirectly. However, they stated that miR-101-3p targets the 3'-UTR of BIM mRNA directly, resulting in increased BIM expression in serum-deprived endothelial cells. In general, all of these evidences suggest that miR-101 has a distinct cellular function in tumor cells versus normal cells. This microRNA is a tumor-suppressive (pro-apoptotic) function in majority of cancers; however, its function in normal cell can be different (Kim et al., 2017[20]).

This information revealed that miR-101 has an important mediator role in mitochondria-dependent intrinsic apoptosis in AML cells and can regulate expression of cell survival genes such as BIM. Since the intrinsic apoptosis pathway is usually regulated by a balance between pro-apoptotic and pro-survival Bcl-2 family proteins. Therefore, BIM is an important component to initiate the intrinsic apoptosis pathway and can result in cell apoptosis induction in AML. 

In conclusion, it can be demonstrated that miR-101 suppresses the growth of cancer cell by up-regulating pro-apoptotic gene, BIM, in AML. Considering the previous studies, down-regulation of endogenous miR-101 occurs in many different types of cancer. So our findings suggest that miR-101 transfection can compensate for depletion of miR-101 and exert unique effects of tumor suppressive miRNA on its target genes. In general, an up-regulation of BIM, a pro-apoptotic protein of Bcl-2 family occurs. Our findings imply that therapeutic strategies aimed at compensation for miR-101 expression may be beneficial to patients with AML.

## Conflict of interest

The authors do not declare any conflict of interest.

## Acknowledgement

The authors thank the supports of Shahid Sadoughi International Campus of Yazd, Iran. We are grateful to Dr. F. Haghirosadat and A. Javid for their helpful comments and suggestions and Dr. F. Montazeri for her assistance in assessing fluorescent microscopic results.

## References

[1] Altucci L, Clarke N, Nebbioso A, Scognamiglio A, Gronemeyer H (2005). Acute myeloid leukemia: therapeutic impact of epigenetic drugs. Int J Biochem Cell Biol.

[2] Amer MH (2014). Gene therapy for cancer: present status and future perspective. Mol Cell Ther.

[3] Bakhshandeh B, Soleimani M, Hafizi M, Ghaemi N (2012). A comparative study on nonviral genetic modifications in cord blood and bone marrow mesenchymal stem cells. Cytotechnology.

[4] Bao R, Shu Y, Hu Y, Wang X, Zhang F, Liang H (2016). miR-101 targeting ZFX suppresses tumor proliferation and metastasis by regulating the MAPK/Erk and smad pathways in gallbladder carcinoma. Oncotarget.

[5] Baumann V, Winkler J (2014). miRNA-based therapies: strategies and delivery platforms for oligonucleotide and non-oligonucleotide agents. Future Med Chem.

[6] Burnett A, Wetzler M, Löwenberg B (2011). Therapeutic advances in acute myeloid leukemia. J Clin Oncol.

[7] Cammarata G, Augugliaro L, Salemi D, Agueli C, Rosa M, Dagnino L (2010). Differential expression of specific microRNA and their targets in acute myeloid leukemia. Am J Hematol.

[8] Cortez MA, Valdecanas D, Zhang X, Zhan Y, Bhardwaj V, Calin G (2014). Therapeutic delivery of miR-200c enhances radiosensitivity in lung cancer. Mol Ther.

[9] Cross D, Burmester JK (2006). Gene therapy for cancer treatment: past, present and future. J Clin Med Res.

[10] Del Burgo LS, Pedraz JL, Orive G (2014). Advanced nanovehicles for cancer management. Drug Discov Today.

[11] Glaser SP, Lee EF, Trounson E, Bouillet P, Wei A, Fairlie WD (2012). Anti-apoptotic Mcl-1 is essential for the development and sustained growth of acute myeloid leukemia. Genes Dev.

[12] Gogada R, Yadav N, Liu J, Tang S, Zhang D, Schneider A (2013). Bim, a proapoptotic protein, up-regulated via transcription factor E2F1-dependent mechanism, functions as a prosurvival molecule in cancer. J Biol Chem.

[13] Gui T, Shen K (2012). miRNA-101: a potential target for tumor therapy. J Cancer Epidemiol.

[14] Huang F, Lin C, Shi YH, Kuerban G (2013). MicroRNA-101 inhibits cell proliferation, invasion, and promotes apoptosis by regulating cyclooxygenase-2 in hela cervical carcinoma cells. Asian Pac J Cancer Prev.

[15] Huang F, Zhao F, Laing LP, Zhou M, Qu ZL, Cao YZ (2015). Optomizing transfection efficiency of cervical cancer cells transfected by cationic liposomes lipofectamineTM2000. Asian Pac J Cancer Prev.

[16] Husain SR, Han J, Au P, Shannon K, Puri RK (2015). Gene therapy for cancer: regulatory considerations for approval. Cancer Gene Ther.

[17] Jayakumar R, Chennazhi KP, Muzzarelli RA, Tamura H, Nair SV, Selvamurugan N (2010). Chitosan conjugated DNA nanoparticles in gene therapy. Carbohydr Polym.

[18] Ji W, Sun B, Su C (2017). Targeting microRNAs in cancer gene therapy. Genes.

[19] Kawabata T, Tanimura S, Asai K, Kawasaki R, Matsumaru Y, Kohno M (2012). Up-regulation of pro-apoptotic protein bim and down-regulation of anti-apoptotic protein Mcl-1 cooperatively mediate enhanced tumor cell death induced by the combination of ERK kinase (MEK) inhibitor and microtubule inhibitor. J Biol Chem.

[20] Kim J, Lee D, Kim J, Choi S, Park W, Ha K (2017). A miRNA-101-3p / Bim axis as a determinant of serum deprivation-induced endothelial cell apoptosis. Cell Death Dis.

[21] Konno Y, Dong P, Xiong Y, Suzuki F, Lu J, Cai M (2014). MicroRNA-101 targets EZH2, MCL-1 and FOS to suppress proliferation, invasion and stem cell-like phenotype of aggressive endometrial cancer cells. Oncotarget.

[22] Liu N, Zhang L, Wang Z, Cheng Y, Zhang P (2017). MicroRNA-101 inhibits proliferation , migration and invasion of human glioblastoma by targeting SOX9. Oncotarget.

[23] Llambi F, Green DR (2011). Apoptosis and oncogenesis: Give and take in the BCL-2 family. Curr Opin Genet Dev.

[24] Luo C, Merz PR, Chen Y, Dickes E, Pscherer A, Schadendorf D (2013). MiR-101 inhibits melanoma cell invasion and proliferation by targeting MITF and EZH2. Cancer Lett.

[25] Luo L, Zhang T, Liu H, Lv T, Yuan D, Yao Y (2012). MiR-101 and Mcl-1 in non-small-cell lung cancer: Expression profile and clinical significance. Med Oncol.

[26] Marcucci G, Haferlach T, Döhner H (2011). Molecular genetics of adult acute myeloid leukemia: prognostic and therapeutic implications. J Clin Oncol.

[27] Nordling-David MM, Golomb G (2013). Gene delivery by liposomes. Isr J Chem.

[28] Peng Y, Croce CM (2016). The role of MicroRNAs in human cancer. Signal Transduct Target Ther.

[29] Plötz M, Gillissen B, Quast SA, Berger A, Daniel PT, Eberle J (2013). The BH3-only protein Bim overrides Bcl-2-mediated apoptosis resistance in melanoma cells. Cancer Lett.

[30] Schnittert J, Kuninty PR, Bystry TF, Brock R, Prakash J (2017). Anti-microRNA targeting using peptide- based nanocomplexes to inhibit differentiation of human pancreatic stellate cells. Nanomedicine (Lond).

[31] Soriano A, Jubierre L, Almazán-Moga A, Molist C, Roma J, De Toledo JS (2013). microRNAs as pharmacological targets in cancer. ‎Pharmacol Res.

[32] Su H, Yang JR, Xu T, Huang J, Xu L, Yuan Y (2009). MicroRNA-101, down-regulated in hepatocellular carcinoma, promotes apoptosis and suppresses tumorigenicity. Cancer Res.

[33] Wang H, Jiang Y, Peng H, Chen Y, Zhu P, Huang Y (2015). Recent progress in microRNA delivery for cancer therapy by non-viral synthetic vectors. Adv Drug Deliv Rev.

[34] Xu L, Beckebaum S, Iacob S, Wu G, Kaiser GM, Radtke A (2014). MicroRNA-101 inhibits human hepatocellular carcinoma progression through EZH2 downregulation and increased cytostatic drug sensitivity. J Hepatol.

[35] Zhang J, Guo JF, Liu DL, Liu Q, Wang J (2011). MicroRNA-101 exerts tumor-suppressive functions in non-small cell lung cancer through directly targeting enhancer of zeste homolog 2. J Thorac Oncol.

